# Plantar cutaneous afferents influence the perception of Subjective Visual Vertical in quiet stance

**DOI:** 10.1038/s41598-018-33268-3

**Published:** 2018-10-08

**Authors:** A. Foisy, Z. Kapoula

**Affiliations:** IRIS team, Physiopathologie de la Vision et Motricité Binoculaire, FR3636 Neurosciences CNRS, Université Paris Descartes, 45 rue des Saints-Pères, 75006 Paris, France

## Abstract

The estimation of Subjective Visual Vertical (SVV) involves the allocentric, gravitational and egocentric references, which are built by visual, vestibular and somatosensory afferents. Our goals were to assess the influence of plantar cutaneous afferents on the perception of SVV, and to see if there is a difference according to the efficiency of plantar cutaneous afferents. We recruited 48 young and healthy subjects and assessed their SVV and postural performances in quiet stance with a force platform, at 40 or 200 cm, in four ground conditions: on firm ground, on foam, with a bilateral, or with a unilateral 3 mm arch support. We also assessed the efficiency of our subjects’ plantar afferents with the plantar quotient method and divided them in two groups: subjects with a normal use of plantar afferents and subjects with Plantar Exteroceptive Inefficiency (PEI). The results showed significant decreases in the counter clockwise SVV deviation only with the unilateral arch support, at near distance, and among the typically behaving subjects. We conclude that asymmetric foot cutaneous afferents are able to bias the egocentric vertical reference and hence influence the perception of SVV. This influence disappears among subjects with PEI, probably because of a distortion of the plantar signal.

## Introduction

Spatial representations require the integration of visual, vestibular and somatosensory cues, which takes place in various areas of the Central Nervous System (CNS). The involved zones which have been identified so far are: the multimodal vestibular areas^1^, the temporo-occipital, parieto-temporal, parieto-occipital and insular cortex^2–4^, the supra-marginal gyrus^5^, the middle temporal gyrus^6^, the inferior frontal and superior temporal^4^, and the posterolateral thalamus^7,8^. These three sensorial modalities enable knowledge of personal and extra-personal space: position of objects in space, positions and displacements of the body in space and spatial configuration of the body segments. Thus, the vestibular system allows to make the distinction between self-motion and movements of the environment. Since motor performance involves an interaction between body and environment^9^, the availability of spatial reference frames is a prerequisite for the control of posture and movement^10–12^.

Three inter-dependent spatial references contribute to the representation of Subjective Visual Vertical (SVV): the allocentric reference, in which the objects are localized directly through their spatial configuration; the gravitational reference, which is independent from the position of the body and objects and refers to the orientation of the gravitational vector; and the egocentric reference, in which the position of the objects is determined with respect to the body^1^. The visual, vestibular and somatosensory afferents contribute respectively to the elaboration of those three references^7,13,14^, giving rise to the internal models of verticality, with the contribution of top-down influences^8^.

The influence of the visual information on SVV estimation has been widely studied, as concerns the static^15–18^ as well as the dynamic cues^16,19,20^. Vestibular afferents also play a major role in the perception of verticality^21–24^. The influence of somatosensory cues has been underscored by Anastasopoulos *et al*.^25^, who showed that two patients with complete hemisensory loss were insensitive to the deviation of SVV estimate (opposite to the side of the body tilt, i.e. A-effect) when lying on the hypoesthetic side. These authors concluded that the A-effect is primarily somatosensory, not otolithic. More recently, other authors^7,26^ obtained similar results among larger groups of paraplegics and hemiplegics with unilateral somatosensory loss. They concluded that somatosensory information plays an important role in the construction and update of internal models of verticality. Likewise, it is also known that patients with cerebral lesions show a spontaneous deviation of their SVV estimates. Such deviation (contralateral to the lesion) occurs only in case of hemispatial neglect and is significantly related to the severity of the neglect^3,6,27,28^. More specifically, the influence of muscular proprioception has been shown by several authors^29–32^. Visceral afferents also play a role^23,33^, as well as tactile cues^33,34^.

As concerns the latter input, it is known that the foot sole behaves like a “dynamometric map” that quantifies the repartition of plantar pressures^35^. According to those authors, plantar pressure distribution is a precious clue that the Central Nervous System uses to assess the importance of the deviation of the body relative to its vertical reference. Furthermore, Roll *et al*.^36^ showed that vibrations applied to the foot soles of restrained and blindfolded subjects give rise to illusory perceptions of whole-body leaning. This result suggests that foot cutaneous afferents contribute to the spatial representation of body posture. However, the existing literature concerning the effects of tactile afferents on SVV only involves the stimulation of body skin during large body tilts (>45°) of seated subjects. To our knowledge, only Faralli *et al*.^37^ studied the influence of the podal input on the perception of SVV, but it was among patients with vestibular dysfunction and by stimulating exteroceptive and proprioceptive afferents at the same time. The specific influence of foot cutaneous afferents on the perception of verticality during quiet standing in healthy subjects has never been studied.

Our main hypothesis (hyp.1) was that plantar cutaneous afferents can influence the perception of SVV. In order to manipulate these afferents we resorted to the interposition of a foam pad, which is known to decrease their availability^38^. Hence, we expected it would increase the deviation in the SVV estimation. We also used bilateral medial arch supports^39^, which are known to improve the quality of postural control^40^. We expected that such stimulation would reduce the deviation of the SVV estimate. Finally, we tested a unilateral (right) lateral arch support, which is supposed to induce an opposite (left) shift of the Center of Pressure^41^ (CoP). Consequently, we expected it would produce an E-effect that is, an error in the SVV estimation opposite to the body tilt^42,43^.

In a previous publication^44^, we noticed that among young and healthy people, there was important inter-individual variability as concerns the effects of thin plantar inserts on postural and oculomotor control (see^40^). We showed that this variability can be explained by the degree to which subjects make use of their plantar afferents. Since plantar cutaneous afferents are normally used by the CNS to ensure postural control^45^, a decrease of their use (especially among healthy subjects) does not seem to correspond to a simple idiosyncrasy.

Hence, we concluded that this minority of healthy people were unable to properly use their plantar cutaneous afferents both for balance and vergence control. We proposed that it corresponds to a situation revealing inefficiency in plantar cutaneous afferents (Plantar Exteroceptive Inefficiency - PEI). We discussed that PEI could be due to a latent somatosensory dysfunction of the sole’s mechanoreceptors linked to an increase in pressure beneath certain plantar zones (such as the first metatarsal head, as proposed by Janin^46^). Knowing that an increase in pressure on the mechanoreceptors increases the discharge frequency of these receptors^47,48^, we argued that such distortion of the plantar signal makes is more difficult to integrate for the CNS^49^, thereby leading to neglect them. These first results were confirmed by another experiment^50^: using the same method, we showed that subjects with PEI were also unable to use plantar and visual afferents for postural control at the same time (visual-podal asynergy).

Here and in our prior publications^44,50^ our view is that, as concerns functional pathology, there is a progressive transition from physiology towards pathology through an in-between dysfunctional state which is not necessarily symptomatic. Our opinion is in line with earlier studies: according to Canguilhem^51,52^, there is, for each function, a margin which corresponds to functional adaptability. He considers physiology as an ability to adapt to the changes of one’s environment, and disease as a reduction to the margin of tolerance of the variations of the environment. Winter^53^ shares the same view specifically as concerns postural control, considering that the adaptability of the CNS explains that pathology may not be apparent at first.

Our previous experiments clearly show that all of the healthy subjects do not use their plantar cutaneous afferents in the same way, and this should be taken into consideration when studying subjects in standing posture. Consequently, we further hypothesized (hyp.2) that PEI would also preclude the influence of foot tactile afferents on the perception of SVV.

## Results

### Group constitution

We divided our population into two groups: the Plantar Exteroceptive Inefficient Subjects, who had a plantar quotient below 100 (21 subjects), and the typically behaving subjects, who had a plantar quotient higher than 100 (27 subjects).

Mann-Whitney U tests showed that subjects with PEI and typically behaving subjects did not have significantly different ages (z = 0.12, *p* = 0.90), heights (z = −0.03, *p* = 0.98), weights (z = 0.58, *p* = 0.65), stereoacuity (z = 0.90, *p* = 0.37), visual acuity (z = −0.22, *p* = 0.83), amplitude of accommodation (z = 1.83, *p* = 0.07) and Near Convergence Point (z = −0.81, *p* = 0.42). Their only significant difference was their plantar quotient (z = −5.89, *p* < 0.01), the subjects with PEI having a lower plantar quotient than the typically behaving subjects, due to a higher Surface area on firm ground (z = 2.28, *p* = 0.02, *d* = 0.82).

### Conditions comparisons

#### Effects of the ground conditions

**Figure 1:**
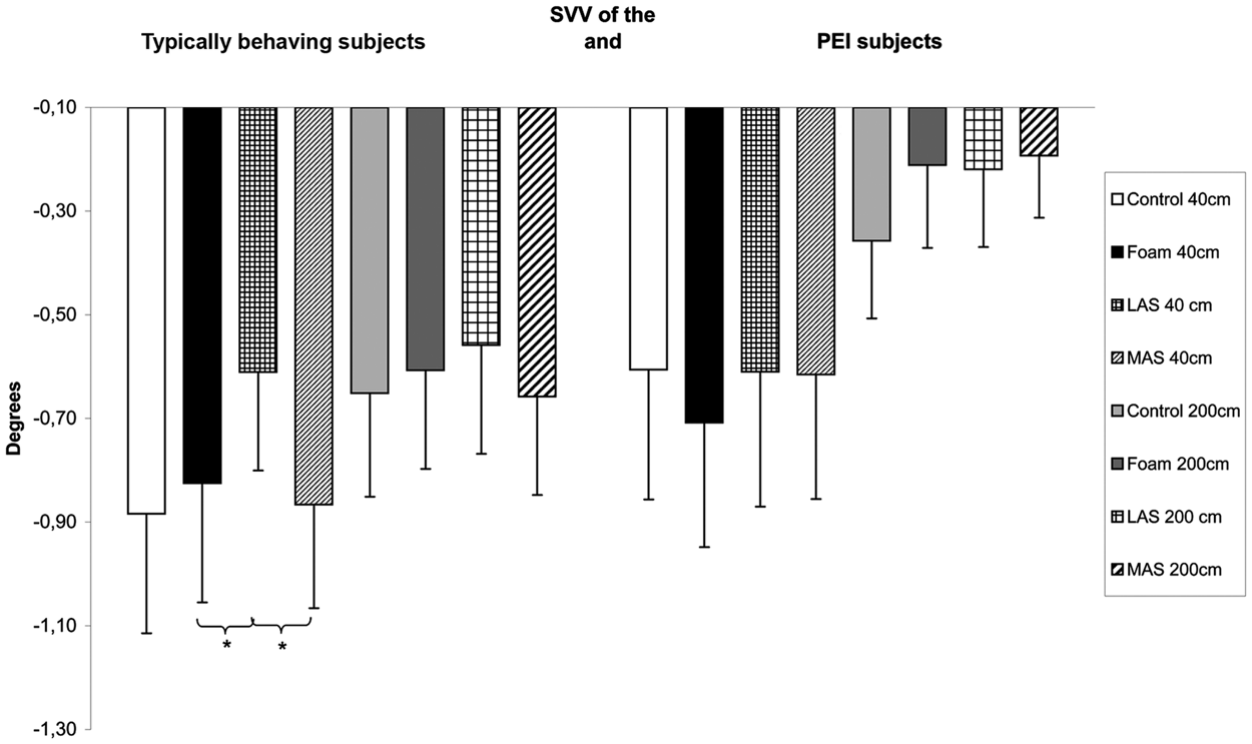
SVV estimation among subjects with Plantar Exteroceptive Inefficiency and typically behaving subjects for each testing condition. Error bars represent the standard errors. Asterisks indicate significant differences, with P with *p < 0.05.

For the typically behaving subjects, the Friedman’s test showed a main effect on the deviation of the SVV (**χ**²_(7,27)_ = 17.64, *p* = 0.01). The SVV was significantly less tilted on the left with the lateral arch support compared to foam (z = 2.45, *p* = 0.04, *d* = 0.19) and to the medial arch supports (z = 2.55, *p* = 0.04, *d* = 0.26). There also was a tendency for the SVV estimate to be less tilted on the left with the lateral arch support compared to Control (z = 1.90, *p* = 0.06, *d* = 0.25) at 40 cm (Fig. 1).

**Figure 2:**
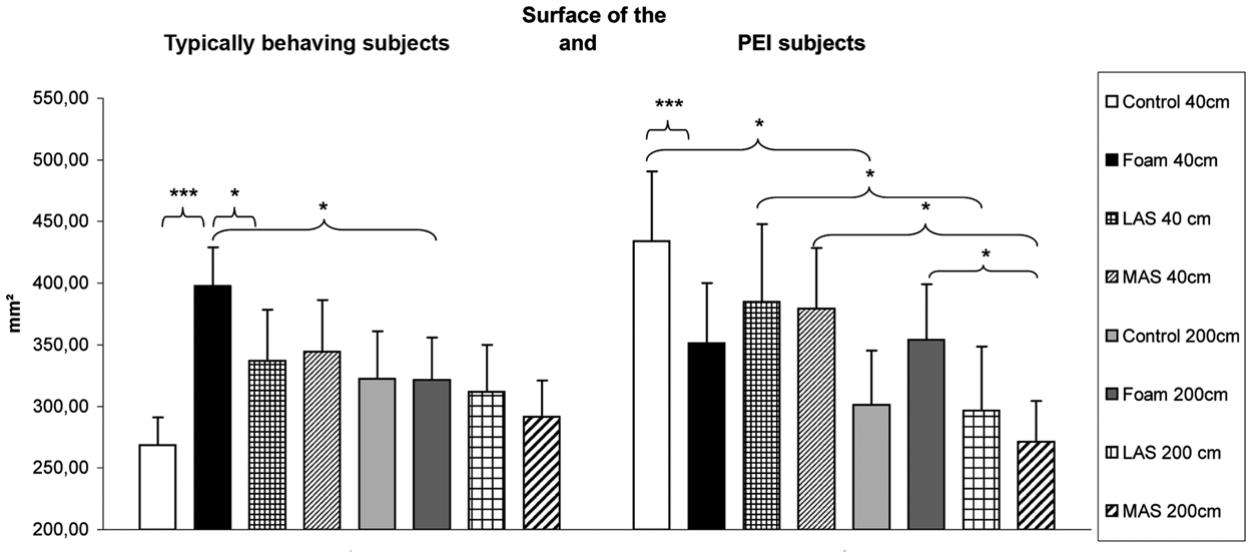
Surface area of the Center of Pressure among subjects with Plantar Exteroceptive Inefficiency and typically behaving subjects for each testing condition. Error bars represent the standard errors. Asterisks indicate significant differences, with P with *p < 0.05, ***p < 0.001.

As concerns postural control, there was a main effect of the ground conditions on the Surface area of the CoP (**χ**²_(7,27)_ = 20.28, *p* = 0.01). At 40 cm, the Surface area was significantly higher on foam compared to firm ground (z = 4.54, *p* < 0.01, *d* = 0.91) and lateral arch support (z = 2.21, *p* = 0.03, *d* = 0.32) (Fig. 2).

**Figure 3:**
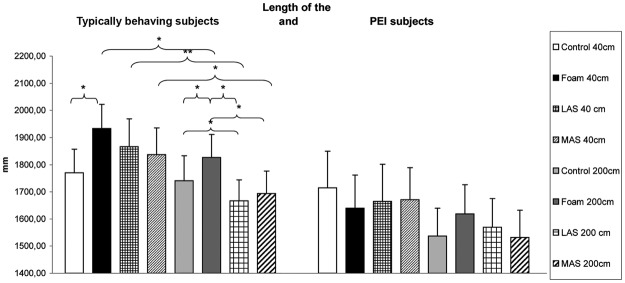
Length of the Center of Pressure excursions for the subjects with Plantar Exteroceptive Inefficiency and typically behaving subjects for each testing condition. Error bars represent the standard errors. Asterisks indicate significant differences, with P with *p < 0.05, **p < 0.01.

There also was a main effect on the Length of the displacements of the CoP (**χ**²_(7,27)_ = 27.38, *p* < 0.01). At 40 cm, the Length of the typically behaving subjects was significantly higher on foam compared to firm ground (z = 3.00, *p* = 0.04, *d* = 0.36), and had a tendency to be higher on foam versus lateral arch support (z = 1.87, *p* = 0.06, *d* = 0.13). At 200 cm, the Length was higher on foam compared to firm ground (z = 2.11, *p* = 0.03, *d* = 0.19), lateral arch support (z = 3.58, *p* = 0.01, *d* = 0.38) and medial arch supports (z = 3.17, *p* = 0.02, *d* = 0.31). It was lower with lateral arch support compared to firm ground (z = 2.04, *p* = 0.04, *d* = 0.17) (Fig. 3).

There was no significant effect on the medio-lateral (X) position of the CoP (**χ**²_(7,27)_ = 3.61, *p* = 0.82).

For the subjects with PEI, there was no significant effect of the ground conditions on the estimation of the SVV (**χ**²_(7,21)_ = 11.47, *p* = 0.12) (Fig. 1).

As concerns postural control, there was a main effect on the Surface area of the CoP (**χ**²_(7,21)_ = 25.74, *p* < 0.01). At 40 cm, the Surface area was lower on foam compared to firm ground (z = 4.01, *p* < 0.01, *d* = 0.34), contrary to the typically behaving subjects. At 200 cm, the Surface area was also lower with medial arch supports compared to foam (z = 2.35, *p* = 0.04, *d* = 0.46) (Fig. 2).

Finally, there was no significant effect concerning the Length of the CoP displacements (**χ**²_(7,21)_ = 6.06, *p* = 0.53 – Fig. 3), and the medio-lateral (X) position of the CoP (**χ**²_(7,21)_ = 4.76, *p* = 0.69).

#### Effects of distance

The results also showed effects of distance. Among the typically behaving subjects, there was a tendency for the SVV estimate to be less tilted on the left at 200 cm than 40 cm on firm ground (z = 1.80, *p* = 0.07, *d* = 0.21), and with medial arch supports (z = 1.78, *p* = 0.08, *d* = 0.21). The Surface area was significantly lower at 200 cm than 40 cm on foam (z = 2.28, *p* = 0.04, *d* = 0.45). The Length was lower at 200 cm than 40 cm on foam (z = 2.39, *p* = 0.02, *d* = 0.24), with the lateral arch support (z = 2.91, *p* < 0.01, *d* = 0.42) and the medial arch supports (z = 2.14, *p* = 0.03, *d* = 0.30).

Among the subjects with PEI, the Surface area was significantly lower at 200 cm than 40 cm on firm ground (z = 2.97, *p* = 0.05, *d* = 0.57), with lateral arch support (z = 2.07, *p* = 0.04, *d* = 0.33), and medial arch supports (z = 2.42, *p* = 0.05, *d* = 0.56).

The results are summarized in Figs 2 and 3 and Table 1.

**Table 1:** SVV and postural performances.

	SVV (degrees)	Surface area of CoP (mm²)	Length of CoP (mm)	PEI subjects	Typically behaving subjects	PEI subjects
	Typically behaving subjects	PEI subjects	Typically behaving subjects			
Control						
At 40 cm	−0.88 ± 0.23, [−1.35, −0.42]	−0.61 ± 0.25, [−1.13, −0.08]	269 ± 23, [222, 315]	434 ± 56, [316, 552]	1771 ± 86, [1594, 1947]	1715 ± 136, [1432, 1997]
At 200 cm	−0.65 ± 0.20, [−1.06, −0.24]	−0.36 ± 0.15, [−0.68, −0.04]	322 ± 39, [243, 402]	301 ± 44, [209, 393]	1741 ± 92, [1551, 1930]	1537 ± 102, [1324, 1751]
Foam						
At 40 cm	−0.82 ± 0.23, [−1.31, −0.34]	−0.71 ± 0.24, [−1.20, −0.22]	398 ± 32, [333, 463]	351 ± 49, [249, 453]	1934 ± 89, [1751, 2117]	1640 ± 122, [1384, 1895]
At 200 cm	−0.61 ± 0.19, [−1.00, −0.22]	−0.21 ± 0.16, [−0.54, 0.12]	321 ± 34, [251, 392]	354 ± 45, [260, 448]	1827 ± 84, [1654, 2000]	1619 ± 107, [1396, 1842]
LAS						
At 40 cm	−0.61 ± 0.19, [−1.00, −0.22]	−0.61 ± 0.26, [−1.16, −0.06]	337 ± 42, [252, 423]	385 ± 63, [254, 517]	1867 ± 102, [1656, 2078]	1665 ± 136, [1381, 1949]
At 200 cm	−0.56 ± 0.21, [−0.98, −0.13]	−0.22 ± 0.15, [−0.54, 0.10]	312 ± 38, [233, 390]	297 ± 52, [189, 404]	1667 ± 77, [1508, 1826]	1569 ± 106, [1348, 1790]
MAS						
At 40 cm	−0.87 ± 0.20, [−1.27, −0.46]	−0.62 ± 0.24, [−1.12, −0.11]	344 ± 42, [258, 431]	379 ± 50, [276, 483]	1837 ± 98, [1635, 2039]	1670 ± 119, [1423, 1918]
At 200 cm	−0.66 ± 0.19, [−1.05, −0.26]	−0.19 ± 0.12, [−0.44, 0.06]	292 ± 29, [231, 352]	271 ± 33, [202, 340]	1694 ± 82, [1525, 1863]	1532 ± 101, [1322, 1741]

Means, standard errors and 95% Confidence Intervals of SVV and postural parameters for each condition among the normal and the Plantar Exteroceptive Inefficient subjects.

## Discussion

### Effects of the ground conditions

The main result of that experiment is that foot cutaneous afferents influence the perception of SVV, confirming our first hypothesis (hyp.1). Furthermore, this influence disappears among subjects with PEI, confirming our additional hypothesis (hyp.2).

#### Effects upon SVV

Our subjects had a mean deviation of the SVV (on all of the conditions) −0.60 ± 1.00°, which means they made a counter clockwise error of 0.60 ± 1.00°. These results are in line with the literature: Akin *et al*.^54^ found that in young healthy individuals, the deviation in the SVV estimate was <2°.

Among the typically behaving subjects the unilateral lateral arch support decreases the counter clockwise deviation at close distance, showing that this way of manipulating plantar cutaneous afferents influences the perception of verticality. Given that the unilateral lateral arch support is the only tested stimulation to induce a modification of the repartition of plantar pressures between the two feet (as shown by Janin and Dupui^41^), it seems that this modification biased the egocentric vertical reference of the CNS, resulting in the observed SVV effect. This rationale is in line with the conclusions of Barra *et al*.^55^, who showed a correlation between the SVV estimate and an unequal distribution of loading on the feet among stroke survivors. Our results are also in accordance with those of other authors: they give a first confirmation of the proposition of Kavounoudias *et al*.^35^ that the CNS uses the information of the foot soles’ pressure distribution in order to build an internal model of verticality. In the same way, Anastasopoulos *et al*.^25^ proposed that the egocentric vertical reference (called “idiotropic vector” by Mittelstaedt^56^) would rely on symmetric somatosensory input, thus explaining the absence of A-effect only among patients with hemisensory loss. It is likely that the same mechanism is involved in healthy subjects, with smaller magnitude. Barra *et al*.^7^ and Saeys *et al*.^26^ also showed that the deviation of the SVV estimates of paraplegics and hemiplegics only exists in case of unilateral somatosensory loss and is proportional to the availability of somatosensory afferents. They concluded that there exists an internal model of verticality in human, with a subjective vertical constructed by synthesizing available sensory information. Hence, our results suggest that plantar cutaneous afferents, and most especially the comparison of the symmetry/asymmetry of the signals arising from the two feet, is a part of the sensory information which is used in this process.

This finding does not seem to be the result of an E-effect because there was no significant contra-lateral shift of the CoP with such plantar insert. Such absence of postural result in the frontal plane is surprising because Janin and Dupui^41^ obtained a contra-lateral shift of the CoP with a midfoot 3mm-thick plantar insert. In their experiment, however the subjects were looking straight ahead in a normally lit room, whereas in this one they were in the dark and were instructed to focus on the verticalisation of the laser. Thus, in this double-task paradigm, the Central Nervous System had to manage the SVV estimation and postural control at the same time, without visual afferents. Such challenging situation may explain the prioritization on the adjustment of the SVV and therefore, that absence of postural regulation in the frontal plane.

Contrary to the typically behaving subjects, the subjects with PEI do not show any modification in the SVV deviation with the lateral arch support. Previous work suggested that PEI is due to a non-noxious somatosensory dysfunction which prevents the CNS from correctly processing and using foot cutaneous afferents for both balance and vergence control^44^. An increase in the pressure beneath the first metatarsal head leading to an increase in the frequency discharge of the sole receptors is thought to be responsible for this dysfunction^44,46,50^. Thus, the results of this study are in line with the former and show that the presence of PEI also precludes plantar cutaneous afferents from influencing the SVV estimates.

#### Effects on postural control

The results concerning posture with the thin plantar inserts (lateral arch support and medial arch supports) showed an improvement of the quality of postural control among typically behaving subjects, through a decrease in Surface area and Length compared to the foam or firm ground condition. These findings are in agreement with previous work of our team^40,44^, even though the postural effects of the medial arch supports were smaller in this experiment. Likewise, it can be due to the double-task here asked for, and the longer recording of postural control (180 seconds), while in the cited experiments the subjects were only looking straight ahead or were performing vergence movements for shorter periods (51 seconds).

The observed effects of improvement of stability with the inserts among typically behaving subjects almost entirely disappear among subjects with PEI. They do not show any modification of the Length of their CoP displacements; the only persisting effect is an improvement of stability as indicated by the decrease in the Surface area with the medial arch supports compared to foam at 200 cm. Furthermore, in agreement with previous results^44,50^, the two groups behave differently on foam: it renders the typically behaving subjects more unstable (increase in Surface area and Length as compared to firm ground), whereas it makes the subjects with PEI more stable in terms of a decrease in their Surface area. Foam smoothes plantar pressure distribution^57^ and decreases the plantar signal^38^. Hence, it appears logical that the decrease in the normal foot cutaneous afferents of the typically behaving subjects results in an increase in body sway among them, whereas the decrease in the excessive and disruptive plantar signal of the subjects with PEI makes them more stable. These results are also in line with previous work of our group^44,50^: they strengthen the hypothesis that, even non noxious, PEI distorts the plantar signal which prevents the affected subjects from properly using these afferents for postural control.

Interestingly, foam interposition produced the expected effects on balance, but no significant effect on SVV. It is known that foam interposition decreases the availability of plantar cues^38,44,58^ which results in a less stable balance. It is confirmed here by the increase in the Length as compared to the 3 other conditions among the typically behaving subjects. Our results are in line with those of Faralli *et al*.^37^ who did not show any effect on the SVV deviation during the perturbation of the podal input of healthy subjects (control group) with a cushion between the feet and the ground. However, these authors did not report the exact characteristics of the cushion (thickness, density, hardness), making impossible to know if it exerts its action rather on plantar exteroception or proprioception^44,58^.

Thus, the postural and SVV results with all of the stimulations (medial arch supports, lateral arch support, foam) seemed independent, confirming previous work^59^. It suggests that the influence of plantar afferents on the multimodal vestibular areas involved in SVV estimation^1,2^ are rather direct than indirect (i.e., following postural changes). Those findings are in line with our previous results concerning vergence and posture^40^. It is worth noting that we only recorded the displacements of the CoP, but it has been shown that force plates’ measurements reflect the same properties of movements as actual body motions in quiet stance (especially for the hip, shoulder and head^60^).

### Effects of distance

Firstly, it appears that the effects of lateral arch support on SVV appear only at close distance (40 cm), that is, when the proprioceptive extra ocular signals are the most available due to the increase in the vergence angle^61^. Hence, given that foot cutaneous afferents play a role in the perception of SVV only for that condition (physiologically, for typically behaving subjects), it seems that those cues need to be compared to the eye muscle afferents to produce their effect. These findings suggest that eye muscle proprioception and foot plantar afferents both take part in the elaboration of the egocentric reference and operate in a synergic way in order to estimate verticality, as they do to ensure balance^50^.

Second, as concerns the control of posture, manipulation of foot tactile afferents produces its effects mainly at far distance (200 cm). It can be explained by the fact that, for postural control, the increase in distance provokes a decrease in the use of visual and oculomotor cues in favour of somatosensory afferents^61^.

Finally, balance is not significantly improved at close distance and is even of better quality (i.e. lower Surface area and Length) at far distance. At near distance the angular size of the vertical line was larger for geometrical reasons, therefore causing more postural instability than at far distance, in accordance with the original visual interpretation of Paulus *et al*.^62^. This result is not in opposition with previous studies of our team showing better stability at near than at far distance^50,63^. Indeed, these studies did not include such an active task, i.e. exposure to a misaligned vertical line and active effort to judge its verticality. Nevertheless, the present study does confirm that postural control is dependent on distance and interacts with the type of task that the subject is performing. Further research with objective eye movement recording would be of interest to understand better eye movement behavior and postural control at far versus near during the SVV estimation.

## Conclusion

This study brings initial evidence of the direct influence of foot cutaneous afferents on the perception of SVV and suggests that they participate in the elaboration of the egocentric reference in synergy with eye muscle proprioception. Moreover, this influence is present only among typically behaving subjects, i.e. who are not affected by PEI.

This experiment is a first step in which we tried different ways of manipulating plantar cutaneous afferents, either attenuating them by foam interposition, or increasing them beneath both feet with the bilateral medial arch supports, or increasing them only beneath one foot with the lateral arch support. The key finding is that only the unilateral stimulation produces an effect on the SVV estimation, suggesting that the symmetry of plantar signals arising from the two feet is information that the CNS uses in order to elaborate an internal model of verticality and assess SVV. In order to confirm those first results, further experiment should focus on protocols aiming at attenuating or stimulating plantar cutaneous afferents in an asymmetrical way.

Those results may have clinical implications. Indeed, it has been showed that elderly fallers^64,65^, and subjects suffering from scoliosis^66^, chronic neck pain^67^, or cerebral lesions inducing visuospatial neglect^3,6,27,28^ have a more altered perception of SVV than healthy subjects. Insoles with thin plantar inserts could be a cheap and simple mean to help those patients in order to improve their quality of SVV estimation. Further research is required to confirm this hypothesis and assess the potential long term effects.

## Methods

### Subjects

Forty-height healthy young subjects took part in the study. They were recruited from paramedical schools, 21 males and 27 females, mean age 25 ± 3,3 years, mean height 170.1 ± 8.6 cm, mean body weight 63.7 ± 10.5 kg. Their characteristics are summarized in Table S1 (Supplementary Information).

None of them were taking medication and all of them were asymptomatic. All subjects were emmetropic and wore no glasses. Their visual acuity at close distance was examined by means of Parinaud’s reading test. The results were all normal (2 for 47 subjects, 3 for one of them). Binocular visual function was also assessed with the stereoacuity TNO test and all values were normal, that is 60” of arc or lower. We also measured the Near Convergence Point, which was 5.06 ± 1.82 cm, and the amplitude of accommodation with the push-up method^68,69^ (we did a mean of 3 measures for both tests). The subjects had a mean of 9.37 dioptres (±1.85), which is within Duane’s normative data (9.5 ± 2 dioptres^68^). The t-test test did not show any statistical difference with that theoretical physiologic value (*p* = 0.616).

### Experimental device

#### SVV assessment

The subjects were asked to stand still, barefoot on a force plate. Their perception of the SVV was assessed thanks to the Perspective System (Subjective Vertical and Horizontal v.2.0, produced by FRAMIRAL). It consists of a laser which is projected on the wall in front of the subject, in a dark room in order to avoid visual landmarks^15,33,70^. The upper end of the laser began tilted left (i.e. counter-clockwise) or right (i.e. clockwise), 15 or 30 degrees, in a random order^14,33^ and then turned clockwise or counter-clockwise at a speed of 2.5°/s. The examiner held a remote control and stopped the movement of the laser when the subject advised him. The subjects were able to adjust the position of the laser by telling the examiner to turn it “left” or “right” with the remote control, until they felt it corresponded to the vertical position and said “OK”. The examiner recorded the deviation given by the Perspective System device with a precision of 0.01°. The procedure was repeated 10 times for each ground condition and a mean of those 10 measures was calculated^7,66,70^. The subjects were never informed of the results in order to avoid a corrective feedback^33,43^.

#### Posture assessment

At the same time the force platform (produced by TechnoConcept, Céreste, France, and using the Standards of the Association Française de Posturologie) assessed the postural performances of our subjects. It consists of two clogs placed side by side; forming a 30° angle with heels separated by 4 cm. Each clog holds 2 strain gauges (one beneath the metatarsal heads, one beneath the heel) which are force - electric tension transducers. The height and weight of the subjects were factored into the calculations of the Center of Pressure’s displacements, which were recorded over a period of 180 s. The equipment contained an Analogue – Digital converter of 16 bits and the sampling frequency of the CoP was 40 Hz. We analyzed the following postural parameters: the medio-lateral (X) mean position of the CoP, the Surface area of CoP excursions and the Length of CoP displacements (total length of the path of the CoP in millimeters). The Surface area of the CoP represents 90% of the instantaneous positions of the CoP included within the confidence ellipse, eliminating the extreme points^71^.

#### Testing conditions

**Figure 4:**
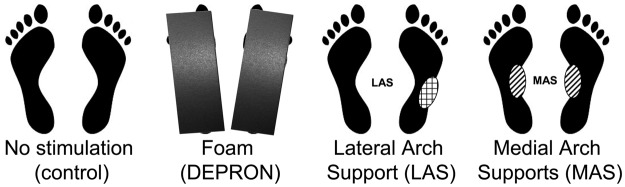
Plantar stimulation conditions. DEPRON foam (6 mm-thick, shore rating of 20A, density of 33 kg/m^3^), lateral arch support, or medial arch supports (3 mm-thick inserts shore rating of 60A, density of 250 kg/m^3^) were placed beneath the feet.

There were 4 plantar stimulation conditions: [1] on firm ground (control condition); [2] on foam (DEPRON, 6 mm-thick, shore rating of 20A, density of 33 kg/m^3^); [3] with a lateral arch support beneath the right foot; [4] with bilateral medial arch supports. The arch supports were 3 mm-thick plantar inserts, made of polyester resin, shore rating of 60A, density of 250 kg/m^3^. The lateral arch support was placed beneath the lateral half of the external mid-foot print, whereas the medial arch supports were set beneath the medial half of the external mid-foot print (see Fig. 4).

It has previously been shown that plantar cutaneous afferents can be experimentally manipulated by mechanical means, either attenuating, or stimulating. The attenuation of the information arising from the feet can be obtained by foam interposition between the ground and feet. Among others, Chiang and Wu^57^, Patel *et al*.^58,72^, Yi and Park^38^ all concluded that foam interposition makes the subjects less stable. In particular, Patel *et al*.^72^ showed that such method produced even stronger disrupting effects on stability than hypothermic anesthesia of fast adapting mechanoreceptors of the foot sole. Furthermore, Yi and Park^38^ directly showed that when healthy subjects are standing on foam, it induces a decrease in plantar cutaneous sensitivity to microfilament touch, similar to the one shown by patients suffering from peripheral sensory neuropathy.

As concerns stimulation of plantar cutaneous afferents, it can be achieved by several means. Plantar exteroceptive signal can be enriched by mechanical vibration of the foot sole, which produces whole-body tilts, opposite to the stimulated foot zone^35,45^. But postural effects can also be obtained with thin plantar inserts set beneath the feet: clinicians^39^ proposed that the keen sensibility of plantar mechanoreceptors (10 mN in pressure, 5 microns in deformation^47,73^ enables them to detect the increase in pressure and deformation of the plantar skin induced by such inserts. Later, Janin and Dupui^41^ confirmed that they can produce similar effects to those obtained by Kavounoudias *et al*.^35,45^. Previous work of our group also showed that such thin plantar inserts significantly improve postural stability^40,44^.

After a first familiarization trial, the postural and SVV estimation performances of each subject were recorded for each plantar condition at close distance (40 cm) and far distance (200 cm), so that there were in total 8 counterbalanced testing conditions. In order to avoid a phenomenon of habituation of the sole cutaneous mechanoreceptors, a one minute period of seated rest separated each recording^74^. We tested the effects of the plantar stimulations at close and far distance because it is known that the CNS assigns a different weight to the signals used in postural control according to the distance of the visual target^61^. At near distance (40 cm) visual and oculomotor signals are particularly used because of the larger retinal slip and vergence angle (respectively); whereas at intermediate and far distances (beyond 90 cm) the CNS mostly uses somatosensory signals^61^. Thus, testing both distances allowed us to see if the same sensory re-weighting is involved in the estimation of SVV.

### Plantar quotient assessment

Thanks to the postural recordings we were able to calculate the plantar quotient^75^. The plantar quotient is a classical method, used by several authors in order to to assess the weight of plantar input in postural control^75–79^. It consists in the ratio between the Surface area of the CoP excursions while the subjects stand on foam and the Surface area while they stand on firm ground: plantar quotient = S_foam_/S_firm ground_ × 100. Hence, the plantar quotient provides information on the weight of plantar cutaneous afferents used in postural control^80,81^: the higher it is, the more the subject relies on the information arising from his feet to keep balance. Indeed, foam decreases the information arising from the feet^38^, normally resulting in a decreased stability^38,44,57,58^, indicated by a plantar quotient >100. We called the subjects exhibiting such typical behavior on foam “typically behaving ubjects”. In contrast, a plantar quotient <100 identifies a subject whose plantar cutaneous afferents impair postural control instead of being useful to balance, thus revealing a Plantar Exteroceptive Inefficiency (PEI^44,50^). In the literature, thick (several cm) and compliant foam support surfaces are generally used, leading to both biomechanical and sensorial effects^38,58^, the latter involving plantar exteroception and proprioception at the same time^57,58^. Here we used thin and firm foam in order to focus the action upon plantar cutaneous afferents (following^44,75,82^). Previous work showed that the plantar quotient is a valuable tool to account for interindividual differences in the use of somatosensory cues and to detect PEI, which is defined by a plantar quotient <100^44,50^.

### Statistical analysis

Statistical analysis was performed using non-parametric tests, i.e Mann-Whitney U tests, Friedman’s tests (procedure of Statsoft/Statistica, release 7.1) since the test of Shapiro-Wilk revealed that some of the distributions were not normal and proved impossible to normalize. Post hoc comparisons were done whenever necessary using the test of Wilcoxon, with *p* < 0.05 considered as significant. The magnitudes of the differences were assessed by the effect size (Cohen’s d).

We applied the Bonferroni-Holm method correction for multiple testing^83,84^, and the corrected p-values are shown in the text.

### Ethics Statement

The investigation adhered to the principles of the Declaration of Helsinki and was approved by the “Conseil d’Evaluation Éthique pour les Recherches en Santé” (CERES) University Paris Descartes, N° IRB: 20153300001072. The subjects gave informed written consent after the nature of the procedure was explained.

## Electronic supplementary material

Subject’s characteristics

Supplementary Dataset 1

## Data Availability

All data generated or analysed during this study are included in this published article (and its Supplementary Information files).
