# Supplementary material for: Plantar cutaneous afferents influence the perception of Subjective Visual Vertical in quiet stance
Source: Sci Rep. 2018 Oct 8;8:14939. doi: 10.1038/s41598-018-33268-3 (PMC6175839; doi:10.1038/s41598-018-33268-3)
Supplement: Supplementary file 1 — Subject’s characteristics [file 41598_2018_33268_MOESM1_ESM.doc]

**Plantar cutaneous afferents influence the perception**

**of Subjective Visual Vertical in quiet stance**

**A. Foisy, Z. Kapoula**

**Table S1.** Subject’s characteristics

| Subject # | Group | PQ | Gender | Age | Height  (cm) | Weight  (kg) | TNO | Parinaud | Accommodation | NCP |
| --- | --- | --- | --- | --- | --- | --- | --- | --- | --- | --- |
| 3 | PEI subject | 42 | M | 27 | 177 | 80 | 30 | 2 | 7,2 | 5,0 |
| 43 | PEI subject | 55 | M | 32 | 165 | 65 | 60 | 2 | 9,7 | 3,0 |
| 16 | PEI subject | 61 | M | 22 | 181 | 80 | 60 | 2 | 14,3 | 6,7 |
| 5 | PEI subject | 65 | F | 26 | 169 | 58 | 15 | 2 | 6,7 | 7,8 |
| 4 | PEI subject | 66 | F | 31 | 159 | 56 | 60 | 2,5 | 9,8 | 6,2 |
| 38 | PEI subject | 72 | F | 25 | 165 | 53 | 60 | 2 | 11,3 | 4,8 |
| 30 | PEI subject | 75 | F | 24 | 176 | 70 | 60 | 2 | 13,0 | 1,0 |
| 12 | PEI subject | 77 | F | 23 | 171 | 53 | 15 | 2 | 9,7 | 4,8 |
| 46 | PEI subject | 78 | F | 21 | 164 | 43 | 30 | 2 | 10,5 | 2,0 |
| 15 | PEI subject | 81 | M | 25 | 166 | 73 | 30 | 2 | 11,5 | 5,3 |
| 21 | PEI subject | 82 | M | 22 | 179 | 68 | 60 | 2 | 10,2 | 5,5 |
| 6 | PEI subject | 82 | F | 25 | 163 | 55 | 60 | 2 | 9,5 | 7,0 |
| 35 | PEI subject | 88 | F | 22 | 147 | 53 | 30 | 2 | 9,0 | 2,0 |
| 31 | PEI subject | 92 | F | 22 | 165 | 62 | 60 | 2 | 8,3 | 1,0 |
| 39 | PEI subject | 92 | F | 22 | 153 | 54 | 60 | 2 | 9,1 | 2,3 |
| 42 | PEI subject | 92 | F | 23 | 162 | 55 | 15 | 2 | 10,0 | 7,2 |
| 11 | PEI subject | 94 | M | 27 | 180 | 84 | 60 | 2 | 12,0 | 6,2 |
| 10 | PEI subject | 95 | M | 33 | 185 | 73 | 60 | 2 | 7,8 | 4,8 |
| 19 | PEI subject | 95 | M | 25 | 183 | 73 | 30 | 2 | 9,4 | 7,7 |
| 24 | PEI subject | 96 | M | 26 | 175 | 70 | 60 | 2 | 7,8 | 4,5 |
| 32 | PEI subject | 98 | M | 24 | 177 | 95 | 30 | 2 | 10,7 | 4,2 |
| *M* |  | 80 |  | 25,1 | 169,6 | 65,4 | 45,0 | 2,0 | 9,9 | 4,7 |
| *SD* |  | 15 |  | 3,4 | 10,1 | 12,9 | 18,4 | 0,1 | 1,9 | 2,1 |
| 23 | Typically behaving subject | 101 | F | 23 | 170 | 56 | 30 | 2 | 9,1 | 6,5 |
| 1 | Typically behaving subject | 102 | M | 20 | 182 | 63 | 30 | 2 | 10,7 | 5,0 |
| 14 | Typically behaving subject | 104 | M | 27 | 185 | 73 | 15 | 2 | 13,3 | 4,8 |
| 2 | Typically behaving subject | 109 | M | 26 | 176 | 84 | 30 | 2 | 7,6 | 7,0 |
| 44 | Typically behaving subject | 110 | M | 24 | 180 | 70 | 60 | 3 | 8,8 | 5,5 |
| 34 | Typically behaving subject | 112 | M | 24 | 176 | 69 | 30 | 2 | 8,2 | 5,0 |
| 8 | Typically behaving subject | 114 | M | 24 | 172 | 64 | 30 | 2 | 9,5 | 4,3 |
| 27 | Typically behaving subject | 114 | F | 31 | 168 | 59 | 30 | 2 | 6,7 | 4,7 |
| 7 | Typically behaving subject | 118 | F | 23 | 165 | 55 | 30 | 2 | 5,9 | 5,3 |
| 45 | Typically behaving subject | 119 | F | 22 | 163 | 52 | 15 | 2 | 8,5 | 3,0 |
| 20 | Typically behaving subject | 121 | F | 21 | 170 | 55 | 30 | 2 | 8,3 | 8,0 |
| 33 | Typically behaving subject | 125 | F | 31 | 160 | 50 | 60 | 2 | 6,3 | 6,3 |
| 40 | Typically behaving subject | 143 | F | 22 | 170 | 65 | 60 | 2 | 7,4 | 2,8 |
| 9 | Typically behaving subject | 147 | M | 26 | 177 | 70 | 30 | 2 | 13,0 | 3,8 |
| 47 | Typically behaving subject | 148 | F | 25 | 162 | 58 | 60 | 2 | 10,2 | 6,3 |
| 41 | Typically behaving subject | 159 | F | 33 | 169 | 63 | 30 | 2 | 6,3 | 5,5 |
| 48 | Typically behaving subject | 168 | F | 27 | 175 | 58 | 60 | 2 | 10,0 | 6,7 |
| 26 | Typically behaving subject | 171 | M | 24 | 178 | 75 | 60 | 2 | 8,6 | 4,0 |
| 28 | Typically behaving subject | 171 | F | 25 | 157 | 59 | 30 | 2 | 10,2 | 2,8 |
| 22 | Typically behaving subject | 189 | M | 26 | 163 | 68 | 30 | 2 | 8,7 | 5,2 |
| 29 | Typically behaving subject | 200 | F | 21 | 169 | 61 | 60 | 2 | 9,4 | 8,2 |
| 25 | Typically behaving subject | 210 | M | 28 | 175 | 68 | 30 | 2 | 9,4 | 6,8 |
| 36 | Typically behaving subject | 224 | F | 21 | 162 | 55 | 60 | 2 | 9,4 | 4,0 |
| 17 | Typically behaving subject | 226 | F | 23 | 174 | 60 | 30 | 2 | 9,1 | 7,5 |
| 13 | Typically behaving subject | 242 | M | 26 | 178 | 68 | 20 | 2 | 8,1 | 4,3 |
| 18 | Typically behaving subject | 252 | F | 21 | 160 | 47 | 60 | 2 | 10,2 | 6,7 |
| 37 | Typically behaving subject | 276 | F | 28 | 166 | 60 | 60 | 2 | 9,1 | 3,7 |
| *M* |  | 158 |  | 24,9 | 170,4 | 62,4 | 39,6 | 2,0 | 9,0 | 5,3 |
| *SD* |  | 52 |  | 3,3 | 7,4 | 8,3 | 16,5 | 0,2 | 1,8 | 1,5 |
| *General M* |  | 124 |  | 25,0 | 170,1 | 63,7 | 42,0 | 2,0 | 9,4 | 5,1 |
| *General SD* |  | 56 |  | 3,3 | 8,6 | 10,5 | 17,3 | 0,2 | 1,8 | 1,8 |

For each subject: stereoacuity (TNO, in seconds of arc), visual acuity at close distance (Parinaud, mean of both eyes), amplitude of accommodation (in dioptres) and Near Convergence Point (NCP, in cm). Normal values are <100 for TNO, <3 for Parinaud and 9.5 ± 2 for the amplitude of accommodation.
